# Twenty‐Five‐Year Alcohol Consumption Trajectories and Their Association With Arterial Aging: A Prospective Cohort Study

**DOI:** 10.1161/JAHA.116.005288

**Published:** 2017-02-20

**Authors:** Darragh O'Neill, Annie Britton, Eric J. Brunner, Steven Bell

**Affiliations:** ^1^ Research Department of Epidemiology and Public Health University College London London United Kingdom; ^2^ Department of Public Health and Primary Care University of Cambridge Cambridge United Kingdom

**Keywords:** aging, alcohol, arterial stiffness, longitudinal cohort study, pulse wave velocity, Epidemiology, Risk Factors, Lifestyle, Cardiovascular Disease, Diet and Nutrition

## Abstract

**Background:**

Emerging evidence suggests that arterial stiffness, an important marker of cardiovascular health, is associated with alcohol consumption. However, the role of longer‐term consumption patterns in the progression of arterial stiffness over time remains unclear. A longitudinal cohort design was used to evaluate the association between alcohol consumption over 25 years and subsequent changes in arterial stiffness.

**Methods and Results:**

Data (N=3869; 73% male) were drawn from the Whitehall II cohort study of British civil servants, in which participants completed repeat pulse wave velocity assessments of arterial stiffness across a 4‐ to 5‐year interval. Repeated alcohol intake measurements were used to categorize participants into alcohol consumer types, accounting for longitudinal variability in consumption. Sex‐stratified linear mixed‐effects modeling was used to investigate whether drinker types differed in their relationship to pulse wave velocity and its progression over time. Males with consistent long‐term heavy intake >112 g of ethanol/week had significantly higher baseline pulse wave velocity (b=0.26 m/s; *P*=0.045) than those who drank consistently moderately (1–112 g of ethanol/week). Male former drinkers showed significantly greater increases in arterial stiffness longitudinally compared to consistently moderate drinkers (b=0.11 m/s; *P*=0.009). All associations were nonsignificant for females after adjustment for body mass index, heart rate, mean arterial pressure, diabetes mellitus, high‐density lipoprotein, and triglycerides.

**Conclusions:**

This work demonstrates that consistently heavy alcohol consumption is associated with higher cardiovascular risk, especially among males, and also provides new insights into the potential impact of changes in drinking levels over time. It discusses the additional insights possible when capturing longitudinal consumption patterns in lieu of reliance on recent intake alone.

**Clinical Trial Registration:**

URL: http://www.clinicaltrials.gov. Unique identifier: NCT02663791.

## Introduction

Cardiovascular diseases remain the leading cause of mortality, accounting for 30% of global deaths.^1^ Research has suggested that moderate levels of alcohol consumption are associated with lower risk of cardiovascular disease onset.^2^ The mechanisms underlying this association are not fully understood nor the impact of changes in drinking levels over time. There is a need to further examine how alcohol consumption can impact on cardiovascular functioning and risk.

Arterial stiffness, which occurs where the vessel wall lacks elasticity, is 1 indicator of cardiovascular health that may be directly affected by alcohol consumption. This stiffness alters arterial responsiveness to pressure variations and is indicative of both functionally and structurally adverse changes.^3^ It is independently linked to both cardiovascular morbidity and mortality, which has been attributed to the impact that arterial stiffness has on hemodynamic processes within the vasculature.^4^, ^5^ The importance of arterial stiffness to cardiac health has led to proposals that it can be used as a surrogate endpoint for studies of cardiovascular disease.^6^ It can be assessed accurately and noninvasively using pulse wave velocity (PWV) estimation.^7^ Arterial waveforms travel faster in less‐elastic vessels, so PWV values are inversely related to such elasticity.^8^

Research into alcohol consumption and PWV^9^, ^10^, ^11^, ^12^ suggests that their association reflects the same U‐/J‐shaped relationship observed elsewhere in cardiovascular‐focused studies of alcohol, implying that moderate alcohol intake may have a protective effect on arterial stiffness.^13^ This pattern has been reflected in cross‐sectional studies of specific population subgroups, such as diabetics and different age groups.^12^, ^13^, ^14^ Multiple mechanisms underlying this association have been proposed, including alcohol‐induced increases in high‐density lipoprotein cholesterol, as well as decreases in platelet adhesiveness to the endothelium.^15^ Conversely, the greater stiffness associated with high consumption may be attributed to an alcohol‐related increase in metalloproteinase activity.^16^

Studies examining associations between alcohol and arterial stiffness have predominantly relied on cross‐sectional data, yet such designs mask longitudinal variability in consumption levels and hinder comparisons of former drinkers to nondrinkers.^2^, ^17^ Moreover, it has been suggested that short‐ and long‐term consumption may have divergent effects on PWV.^18^ New insights could therefore be garnered by investigating longer‐term patterns of alcohol intake that properly capture the impact that variable intake levels and discontinuation of drinking may have on arterial stiffness. Capturing longitudinal measurements of PWV also would help evaluate alcohol's association with progression in arterial stiffness and thus with arterial aging more generally.^4^

Two earlier cohort studies have shown that daily consumption of ≥23 g of ethanol is associated with increased incidence of arterial stiffness over a 9‐year period.^19^, ^20^ A more‐recent study found that consuming alcohol twice‐weekly or more is associated with significant PWV increases across a 5‐year interval.^21^ These 3 studies comprised male participants only and relied on a single cross‐sectional assessment of alcohol consumption. To our knowledge, only 1 study of the association between alcohol and changes in PWV has used repeat assessments of alcohol consumption.^22^ This study found that moderate drinkers with normal blood pressure had significantly smaller increases in PWV over 6 years than did nondrinkers. Heavy drinkers with high blood pressure had significantly greater increases in PWV than their nondrinker or moderate drinker counterparts. The study, however, did not assess whether longitudinal changes in consumption patterns influence these associations.

Using repeat alcohol assessments covering more than 2 decades, we aim, in the current study, to more accurately capture complexity in how drinking behaviors are associated with arterial stiffness than has been previously achieved. The primary aims of this study are to examine whether long‐term patterns of alcohol consumption are independently associated with a baseline assessment of PWV and with longitudinal change in PWV. Secondary aims include an examination of whether short‐term intake levels show comparable associations with PWV.

## Methods

### Sample and Design

**Figure 1:**
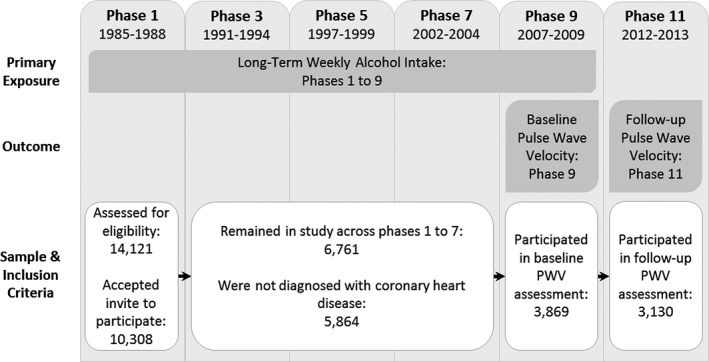
Participant selection flowchart. PWV indicates pulse wave velocity.

The data have been sourced from the Whitehall II cohort study. This study incorporates longitudinal assessments of 10 308 UK civil servants, originally recruited between 1985 and 1988. These recruits comprised 6895 males (67%) and 3413 females, with an overall age range of 34 to 56 years. Arterial stiffness is deemed a surrogate endpoint for cardiovascular disease, as outlined in the introduction in the main article. Consequently, participants with a previous history of cardiovascular disease were excluded from the analytic sample, leading to the exclusion of 897 participants. Clinical assessments were repeated at 4‐ to 5‐year intervals. PWV measurement was introduced to the clinical protocol at phase 9, with assessments taking place in 2007–2009. Three thousand eight hundred sixty‐nine participants had a baseline PWV assessment during this time. Follow‐up testing took place at phase 11, during 2012 and 2013. Of the participants who underwent a PWV assessment at phase 9, 3130 also had a successful PWV assessment at phase 11. The participant selection process is illustrated in Figure 1.

Approval for the Whitehall II study was received from the University College London Medical School Committee on the ethics of human research, and participants gave written informed consent. Whitehall II data are available to bona‐fide researchers for research purposes. The Whitehall II data‐sharing policy is available at http://www.ucl.ac.uk/whitehallII/data-sharing.

### Measures

#### Outcome

The outcome of interest is carotid‐femoral PWV, measured in meters per second (m/s). Research has shown that carotid‐femoral PWV offers a more‐sensitive arterial stiffness measurement than PWV evaluation at other sites,^23^ and it has been described as the “gold‐standard” assessment approach.^2^ Higher PWV values are indicative of increased arterial stiffness.

PWV was assessed using applanation tonometry (SphygmoCor device; Atcor Medical, Sydney, Australia). Path length was ascertained using both the carotid‐sternal notch distance and femoral‐sternal notch distance. Participants underwent up to 3 PWV measurements at each phase to ensure assessment accuracy, and the mean of these was taken as their PWV measurement for that phase.

#### Exposure

Self‐reported alcohol consumption was assessed at phases 1, 3, 5, 7, and 9. Participants reported the number of glasses of wine, pints of beer/cider, and measures of spirit/liqueur consumed in the week preceding each assessment. These values were then converted into ethanol volumes. In terms of conversion ratios, 8 g of ethanol was assumed for each measure of spirit and 16 g for each pint of beer/cider.^24^ The ethanol content of wine was estimated in line with recent guidance regarding the increased alcohol content in a standard wine glass since 1995.^25^, ^26^ Each wine glass consumed pre‐1995 was assumed to contain 8 g of ethanol and 16 g after this date.

Weekly consumption levels for all beverage types combined were then used to categorize intake according to the current UK guidelines.^27^ These define moderate consumption for males and females as weekly ethanol intake volumes up to 112 g (≤14 UK units).

Utilizing these self‐reported weekly ethanol intake data, participants' pattern of long‐term alcohol consumption across phases 1, 3, 5, 7, and 9 was then derived. Definitions and counts for this long‐term intake categorization are presented in Table 1. Unstable drinker types were labeled according to their modal intake level. Further refinement in the categorization of drinker types was limited by statistical power. Where participants were missing consumption data in more than 3 phases, long‐term drinker type could not be reliably determined and, accordingly, long‐term drinker type was deemed missing for 47 participants. Recent consumption patterns, based on reported intake at phase 9 only, were also evaluated as part of supplementary analyses. This alternative categorization and associated counts are again described in Table 1. Recent intake data were missing for 78 participants.

**Table 1:** Long‐Term and Recent Drinker Type Definitions With Observed Counts and Percentages (Within‐Sex and Overall)

	Drinker Type	Weekly Ethanol Intake	N (%)		
			Male	Female	Total
Long‐term drinker type (Phases 1, 3, 5, 7, 9)	Stable nondrinker	0 g at each phase	85 (3.0)	82 (8.2)	167 (15.0)
	Stable moderate drinker	1 to 112 g at each phase	390 (13.8)	179 (17.8)	569 (51.3)
	Stable heavy drinker	>112 g at each phase	500 (17.7)	41 (4.1)	541 (48.7)
	Unstable moderate drinker	>0 g at phase 9 and 1 to 112 g across more than half, but not all, of phases 1 to 9	787 (27.9)	323 (32.2)	1110 (29.0)
	Unstable heavy drinker	>0 g at phase 9 and >112 g across at least half, but not all, of phases 1 to 9	807 (28.6)	191 (19.0)	998 (26.1)
	Former drinker	0 g at phase 9 but intake >0 g at any earlier phase	249 (8.8)	188 (18.7)	437 (11.4)
Recent drinker type (Phase 9)	No recent intake	0 g at phase 9	334 (11.9)	269 (27.0)	603 (15.9)
	Recent moderate	1 to 112 g at phase 9	1110 (39.7)	466 (46.8)	1576 (41.6)
	Recent heavy	>112 g at phase 9	1351 (48.3)	261 (26.2)	1612 (42.5)

N indicates count.

#### Covariates

To account for potential confounds, known demographic and clinical risk factors for arterial stiffness were selected for inclusion in the modeling. The covariates comprised standard demographic characteristics, including age,^28^ sex,^29^ and ethnicity.^30^ Socioeconomic position was also included^31^ and categorized as low, medium, or high according to participants' most recent employment grading. Smoking status was assessed,^32^ with participants categorized as current, former, or nonsmokers. Exercise level was likewise measured.^33^ This was established by determining whether participants' self‐reported activity level met or exceeded 2.5 hours of moderate‐to‐vigorous activity per week, the World Health Organization's (WHO) recommended amount. Clinical covariates included body mass index^34^ and type II diabetes mellitus as determined through the HbA1c blood glucose test.^28^ Mean arterial pressure^35^ was determined using both diastolic and systolic pressures at the time of the PWV assessment. Heart rate,^36^ high‐density lipoprotein cholesterol,^37^ and triglyceride levels^38^ were also measured. Finally, to account for variability in time between the PWV baseline and follow‐up assessments, the time difference was calculated for each participant and standardized to a 4‐year period (because of the mean interval being 4.1 years). The covariates were assessed at the baseline PWV assessment (phase 9, 2007–2009), and mean centering was performed before modeling for those measured on a continuous scale.

### Statistical Analysis

To account for the existing cross‐sectional evidence that sex may moderate the alcohol‐PWV relationship, the analyses in the current study were stratified by participant sex.^13^, ^39^ Linear mixed‐effects modeling was then used to examine the effect of alcohol drinker type on PWV and longitudinal changes in this marker. A series of random intercept models were developed using maximum likelihood estimation,^40^ with long‐term drinker type as the exposure in the first instance. A second set of models was generated with the recent drinker type as exposure. The interval between assessments (standardized to 4 years) was used as the time indicator in all models. The statistical analyses were performed in R (v3.3.0; R Foundation for Statistical Computing, Vienna, Austria). For both the long‐term and recent intake exposures, models were developed iteratively with increasing numbers of covariates included, beginning with age, sex, and assessment interval, followed by demographic and lifestyle factors (ethnicity, smoking, socioeconomic position, and exercise level) and ending with clinical factors (body mass index, type II diabetes mellitus, mean arterial pressure, heart rate, and high‐density lipoprotein cholesterol). The reference category for long‐term consumption was stable moderate drinkers and for the recent intake analysis was moderate intake.^41^

To address missing data, multiple imputation by chained equations was undertaken using the R “mice” package (v2.25; R Foundation for Statistical Computing), with 100 imputations performed for both the male‐only and female‐only data subsets. The outcome variable was included in the imputation model, but not itself imputed; auxiliary variables were also utilized to improve precision and adherence to the missing at random assumption. Sensitivity analyses were undertaken to examine whether complete case analyses produced comparable results.

All tests of statistical significance were 2‐tailed, and a threshold of *P*<0.05 was used for inferring significance.

## Results

Of the total 3869 participants in the analysis sample, 73.7% were male. As shown in Table 1, the male sample had a much higher proportion of heavy drinkers (stable, 17.7%; unstable, 27.9%) than did the female sample. Conversely, there were over twice as many stable nondrinkers (8.2%) and former drinkers (18.7%) in the female cohort as in the male. Unstable moderate drinkers were the most common type among females (32.2%) and were also prevalent among the male sample (27.9%). Sociodemographic and clinical characteristics of the male and female cohorts are provided in Tables 2 and 3, respectively, with additional stratification by long‐term drinking categorization.

**Table 2:** Male Long‐Term Drinker Types at PWV Baseline: Sample Characteristics

Covariate	Subcategory	Long‐Term Drinker Type							Overall (N=2852)
		Stable Nondrinker (N=85)	Stable Moderate (N=390)	Stable Heavy (N=500)	Unstable Moderate (N=787)	Unstable Heavy (N=807)	Former Drinker (N=249)	Unknown (N=34)	
		N (%)	N (%)	N (%)	N (%)	N (%)	N (%)	N (%)	N (%)
PWV (m/s), mean (SD)		8.8 (2.0)	8.3 (2.0)	8.7 (2.0)	8.5 (2.0)	8.4 (1.9)	8.6 (2.1)	8.5 (2.2)	8.5 (2.0)
Age (yr), mean (SD)		65.4 (6.1)	65.1 (5.8)	64.8 (5.4)	65.4 (5.7)	64.7 (5.6)	65.1 (5.5)	63.4 (4.8)	65 (5.6)
Ethnicity	White	60 (70.6)	367 (94.1)	491 (98.2)	744 (94.5)	778 (96.4)	218 (87.6)	32 (94.1)	2690 (94.3)
	South Asian	20 (23.5)	16 (4.1)	2 (0.4)	28 (3.6)	18 (2.2)	16 (6.4)	2 (5.9)	102 (3.6)
	Black	5 (5.9)	6 (1.5)	2 (0.4)	14 (1.8)	4 (0.5)	8 (3.2)	0 (0)	39 (1.4)
	Other	0 (0)	1 (0.3)	3 (0.6)	1 (0.1)	5 (0.6)	5 (2)	0 (0)	15 (0.5)
	Unknown	0 (0)	0 (0)	2 (0.4)	0 (0)	2 (0.2)	2 (0.8)	0 (0)	6 (0.2)
Smoking	Never smoked	58 (68.2)	229 (58.7)	153 (30.6)	406 (51.6)	333 (41.3)	124 (49.8)	10 (29.4)	1313 (46)
	Ex‐smoker	22 (25.9)	137 (35.1)	295 (59)	338 (42.9)	429 (53.2)	108 (43.4)	18 (52.9)	1347 (47.2)
	Current smoker	5 (5.9)	18 (4.6)	44 (8.8)	34 (4.3)	33 (4.1)	17 (6.8)	0 (0)	151 (5.3)
	Unknown	0 (0)	6 (1.5)	8 (1.6)	9 (1.1)	12 (1.5)	0 (0)	6 (17.6)	41 (1.4)
Exercise level	Meets WHO recommendations	24 (28.2)	120 (30.8)	151 (30.2)	233 (29.6)	289 (35.8)	61 (24.5)	8 (23.5)	886 (31.1)
	Does not meet WHO recommendations	61 (71.8)	263 (67.4)	345 (69)	550 (69.9)	510 (63.2)	188 (75.5)	21 (61.8)	1938 (68)
	Unknown	0 (0)	7 (1.8)	4 (0.8)	4 (0.5)	8 (1)	0 (0)	5 (14.7)	28 (1)
Socioeconomic position	Low	11 (12.9)	13 (3.3)	7 (1.4)	35 (4.4)	22 (2.7)	17 (6.8)	2 (5.9)	107 (3.8)
	Medium	46 (54.1)	167 (42.8)	171 (34.2)	327 (41.6)	263 (32.6)	129 (51.8)	17 (50)	1120 (39.3)
	High	28 (32.9)	210 (53.8)	322 (64.4)	425 (54)	522 (64.7)	103 (41.4)	15 (44.1)	1625 (57)
Body mass index (kg/m^2^), mean (SD)		24.9 (3.6)	25.6 (3.3)	26.6 (3.4)	25.8 (3.4)	26.3 (3.4)	26.3 (3.9)	26.5 (4)	26.1 (3.5)
Diabetes mellitus	No	76 (89.4)	356 (91.3)	455 (91)	696 (88.4)	734 (91)	218 (87.6)	31 (91.2)	2566 (90)
	Yes	9 (10.6)	34 (8.7)	45 (9)	91 (11.6)	73 (9)	31 (12.4)	3 (8.8)	286 (10)
Mean arterial pressure (mm Hg), mean (SD)		88.7 (9.4)	90.7 (10.5)	91.4 (10.3)	90.4 (10.1)	90.5 (10.1)	90.4 (10.2)	92.6 (12.8)	90.6 (10.2)
Heart rate (bpm), mean (SD)		62.3 (9.2)	63.2 (11.6)	64.8 (11.7)	63 (10.5)	62.6 (9.9)	64.1 (12.4)	65.3 (11.5)	63.4 (10.9)
High‐density lipoprotein cholesterol (mg/dL), mean (SD)		1.4 (0.4)	1.4 (0.4)	1.6 (0.4)	1.5 (0.4)	1.6 (0.4)	1.4 (0.3)	1.6 (0.4)	1.5 (0.4)
Triglycerides (mmol/L), mean (SD)		1.3 (1)	1.3 (0.8)	1.3 (0.9)	1.2 (0.7)	1.2 (0.7)	1.2 (0.5)	1.3 (0.6)	1.3 (0.7)

bpm indicates beats per minute; N, count; PWV, pulse wave velocity; WHO, World Health Organization.

**Table 3:** Female Long‐Term Drinker Types at PWV Baseline: Sample Characteristics

Covariate	Subcategory	Long‐Term Drinker Type							Overall (N=1017)
		Stable Nondrinker (N=82)	Stable Moderate (N=179)	Stable Heavy (N=41)	Unstable Moderate (N=323)	Unstable Heavy (N=191)	Former Drinker (N=188)	Unknown (N=13)	
		N (%)	N (%)	N (%)	N (%)	N (%)	N (%)	N (%)	N (%)
PWV (m/s), mean (SD)		8.6 (2.5)	7.9 (1.8)	8.3 (2.2)	8.4 (2.1)	7.8 (1.7)	8.3 (1.8)	8.7 (1.6)	8.2 (1.9)
Age (yr), mean (SD)		65.5 (6.2)	64.6 (5.6)	63.3 (5.5)	64.8 (5.5)	64.9 (5.6)	66.2 (6)	64.4 (4.4)	65 (5.7)
Ethnicity	White	49 (59.8)	165 (92.2)	41 (100)	286 (88.5)	189 (99)	158 (84)	12 (92.3)	900 (88.5)
	South Asian	20 (24.4)	5 (2.8)	0 (0)	13 (4)	0 (0)	9 (4.8)	0 (0)	47 (4.6)
	Black	9 (11)	8 (4.5)	0 (0)	20 (6.2)	1 (0.5)	18 (9.6)	1 (7.7)	57 (5.6)
	Other	4 (4.9)	1 (0.6)	0 (0)	3 (0.9)	1 (0.5)	3 (1.6)	0 (0)	12 (1.2)
	Unknown	0 (0)	0 (0)	0 (0)	1 (0.3)	0 (0)	0 (0)	0 (0)	1 (0.1)
Smoking	Never smoked	68 (82.9)	118 (65.9)	15 (36.6)	178 (55.1)	76 (39.8)	112 (59.6)	3 (23.1)	570 (56)
	Ex‐smoker	6 (7.3)	51 (28.5)	21 (51.2)	127 (39.3)	101 (52.9)	68 (36.2)	10 (76.9)	384 (37.8)
	Current smoker	5 (6.1)	2 (1.1)	3 (7.3)	10 (3.1)	12 (6.3)	8 (4.3)	0 (0)	40 (3.9)
	Unknown	3 (3.7)	8 (4.5)	2 (4.9)	8 (2.5)	2 (1)	0 (0)	0 (0)	23 (2.3)
Exercise level	Meets WHO recommendations	15 (18.3)	50 (27.9)	6 (14.6)	91 (28.2)	51 (26.7)	35 (18.6)	3 (23.1)	251 (24.7)
	Does not meet WHO recommendations	65 (79.3)	125 (69.8)	35 (85.4)	228 (70.6)	138 (72.3)	153 (81.4)	10 (76.9)	754 (74.1)
	Unknown	2 (2.4)	4 (2.2)	0 (0)	4 (1.2)	2 (1)	0 (0)	0 (0)	12 (1.2)
Socioeconomic position	Low	37 (45.1)	33 (18.4)	0 (0)	70 (21.7)	12 (6.3)	73 (38.8)	4 (30.8)	229 (22.5)
	Medium	35 (42.7)	93 (52)	17 (41.5)	172 (53.3)	80 (41.9)	92 (48.9)	6 (46.2)	495 (48.7)
	High	10 (12.2)	53 (29.6)	24 (58.5)	81 (25.1)	99 (51.8)	23 (12.2)	3 (23.1)	293 (28.8)
Body mass index (kg/m^2^), mean (SD)		26.4 (5.2)	26.2 (4.5)	27.5 (5.3)	26.2 (4.9)	25.1 (4.5)	27 (5.1)	25.8 (4)	26.2 (4.9)
Diabetes mellitus	No	64 (78)	171 (95.5)	39 (95.1)	281 (87)	180 (94.2)	167 (88.8)	11 (84.6)	913 (89.8)
	Yes	18 (22)	8 (4.5)	2 (4.9)	42 (13)	11 (5.8)	21 (11.2)	2 (15.4)	104 (10.2)
Mean arterial pressure (mm Hg), mean (SD)		87.6 (11.2)	86.7 (11.1)	89 (12.1)	86.7 (10.9)	85.8 (11.9)	87.5 (10.2)	87.3 (8.7)	86.8 (11.1)
Heart rate (bpm), mean (SD)		67.7 (14.5)	64.9 (9.1)	63.8 (7.6)	65.9 (11.3)	64.4 (10.3)	64.3 (9.7)	66.4 (4.9)	65.2 (10.6)
High‐density lipoprotein cholesterol (mg/dL), mean (SD)		1.7 (0.5)	1.9 (0.4)	2 (0.5)	1.9 (0.4)	2.1 (0.5)	1.8 (0.4)	1.7 (0.3)	1.9 (0.5)
Triglycerides (mmol/L), mean (SD)		1.3 (0.9)	1 (0.4)	1 (0.4)	1.1 (0.5)	0.9 (0.5)	1.2 (0.7)	1.1 (0.4)	1.1 (0.6)

bpm indicates beats per minute; N, count; PWV, pulse wave velocity; WHO, World Health Organization.

The majority of the male (94.3%) and female (88.5%) participants were of a white ethnicity. The most common socioeconomic position among females was medium (48.7%) and among males was high (57%). Few among either sex were current smokers, whereas the majority (male, 68%; female, 74.1%) failed to meet the WHO's recommended weekly exercise levels. Among both cohorts, 1 in 10 had type II diabetes mellitus.

Stable nondrinkers, regardless of sex, were the most likely to have never smoked (male, 68.2%; female, 75.4%), whereas stable heavy drinkers had the highest proportion of current smokers (male, 8.8%; female, 7.3%). Former drinkers (male, 75.5%; female, 81.4%) were most likely to get insufficient exercise. For both sexes, diabetes mellitus was most common among nondrinkers.

The mean PWV at baseline for males was 8.5 m/s (SD=2.0), which significantly increased to 9.1 m/s (SD=2.4) by the follow‐up (t(2349)=19.5; *P*<0.001). The female mean was consistently lower, progressing from 8.2 m/s (SD=1.9) at baseline to 8.7 m/s (SD=2.3) by the follow‐up, also a significant increase (t(779)=10.9; *P*<0.001).

Table 4 presents the multilevel modeling results for all long‐term drinker types and for each modeling stage, with the analyses stratified by sex (complete covariate results are available from the first author on request). In an initial model, adjusting for age and assessment interval, consistently heavy drinkers had significantly higher baseline PWV values than the stable moderate drinking reference group (male, b=0.35 m/s; 95% CI=0.08–0.62; *P*=0.012; female, b=0.66 m/s; 95% CI=0.01–1.32; *P*=0.048). Female participants with an unstable pattern of moderate consumption (b=0.46 m/s; 95% CI=0.10–0.82; *P*=0.012) also had higher PWV than those who consistently drank moderately.

**Table 4:** Effect of Long‐Term Drinker Type on Baseline PWV and Longitudinal Changes in PWV: Sex‐Stratified Results

Model	Long‐Term Drinker Type	PWV at Baseline^a^				Change in PWV (Per 4 Years)			
		Males		Females		Males		Females	
		Estimate (95% CI)	*P* Value	Estimate (95% CI)	*P* Value	Estimate (95% CI)	*P* Value	Estimate (95% CI)	*P* Value
Adjusted for age and assessment interval	Stable nondrinker	0.28 (−0.20, 0.75)	0.260	0.26 (−0.27, 0.79)	0.333	0.05 (−0.07, 0.16)	0.417	0.09 (−0.04, 0.21)	0.161
	Stable moderate drinker	Reference		Reference		Reference		Reference	
	Stable heavy drinker	0.35 (0.08, 0.62)	0.012^b^	0.66 (0.01, 1.32)	0.048^b^	≈0.00 (−0.06, 0.07)	0.947	0.01 (−0.15, 0.17)	0.918
	Unstable moderate drinker	0.14 (−0.11, 0.39)	0.268	0.46 (0.10, 0.82)	0.012^b^	≈0.00 (−0.06, 0.06)	0.944	0.03 (−0.06, 0.11)	0.533
	Unstable heavy drinker	0.10 (−0.15, 0.35)	0.431	−0.15 (−0.55, 0.25)	0.470	0.03 (−0.03, 0.09)	0.378	0.03 (−0.07, 0.12)	0.576
	Former drinker	0.17 (−0.16, 0.50)	0.325	0.16 (−0.25, 0.56)	0.455	0.11 (0.03, 0.19)	0.008^b^	0.02 (−0.07, 0.12)	0.615
As above with adjustment for demographics and lifestyle factors^c^	Stable nondrinker	0.13 (−0.36, 0.61)	0.606	0.14 (−0.40, 0.68)	0.611	0.05 (−0.07, 0.16)	0.410	0.09 (−0.04, 0.21)	0.163
	Stable moderate drinker	Reference		Reference		Reference		Reference	
	Stable heavy drinker	0.39 (0.11, 0.66)	0.006^b^	0.73 (0.07, 1.39)	0.029^b^	≈0.00 (−0.06, 0.07)	0.953	0.01 (−0.15, 0.17)	0.910
	Unstable moderate drinker	0.13 (−0.11, 0.38)	0.291	0.44 (0.09, 0.80)	0.015^b^	≈0.00 (−0.06, 0.06)	0.946	0.03 (−0.06, 0.11)	0.546
	Unstable heavy drinker	0.13 (−0.12, 0.38)	0.296	−0.06 (−0.46, 0.35)	0.781	0.03 (−0.03, 0.09)	0.379	0.03 (−0.07, 0.12)	0.596
	Former drinker	0.12 (−0.21, 0.45)	0.478	0.10 (−0.31, 0.52)	0.628	0.11 (0.03, 0.19)	0.009^b^	0.02 (−0.07, 0.12)	0.632
As above with adjustment for clinical factors^d^	Stable nondrinker	0.30 (−0.15, 0.75)	0.191	−0.06 (−0.56, 0.44)	0.813	0.05 (−0.07, 0.16)	0.414	0.08 (−0.04, 0.20)	0.188
	Stable moderate drinker	Reference		Reference		Reference		Reference	
	Stable heavy drinker	0.26 (0.01, 0.52)	0.045^b^	0.42 (−0.18, 1.02)	0.169	≈0.00 (−0.06, 0.07)	0.937	≈0.00 (−0.16, 0.16)	0.995
	Unstable moderate drinker	0.13 (−0.10, 0.36)	0.252	0.28 (−0.05, 0.61)	0.091	≈0.00 (−0.05, 0.06)	0.884	0.02 (−0.06, 0.11)	0.560
	Unstable heavy drinker	0.13 (−0.10, 0.36)	0.260	−0.12 (−0.50, 0.25)	0.523	0.02 (−0.03, 0.08)	0.416	0.03 (−0.07, 0.12)	0.558
	Former drinker	0.09 (−0.21, 0.40)	0.558	−0.06 (−0.43, 0.32)	0.764	0.11 (0.03, 0.19)	0.009^b^	0.02 (−0.07, 0.12)	0.648

PWV indicates pulse wave velocity.

a Unit of PWV is meters per second (m/s).

b Significant *P* values.

c Ethnicity, smoking, exercise, and socioeconomic position.

d Body mass index, heart rate, mean arterial pressure, diabetes mellitus, high‐density lipoprotein, and triglycerides.

Following adjustment for additional demographic, lifestyle, and clinical factors, the PWV of male stable heavy drinkers remained significantly higher at baseline (b=0.26 m/s; 95% CI=0.01–0.52; *P*=0.045). With the female participants, however, the associations for the stable heavy (b=0.42 m/s; 95% CI=−0.18 to 1.02; *P*=0.169) and unstable moderate consumers (b=0.28 m/s; 95% CI=−0.05 to 0.61; *P*=0.091) were no longer statistically significant, although their effect estimates remained larger than that observed with the male stable heavy drinkers.

These models also enabled the evaluation of differences between the long‐term drinker types in terms of the subsequent change in PWV. Among males, regardless of covariates included in the model, former drinkers showed significantly greater increases in PWV over time relative to stable moderate drinkers (maximally adjusted model, b=0.11 m/s; 95% CI=0.03–0.19; *P*=0.009). Interestingly, these former drinkers had shown a large drop in association with baseline PWV following adjustment for lifestyle and clinical characteristics, yet these covariate adjustments had no impact on the magnitude of the longitudinal association observed in this group or any of the long‐term drinker types. With the female participants, no significant longitudinal effects were found, the largest effect estimate in the maximally adjusted model being for the stable nondrinkers (b=0.08 m/s; 95% CI=−0.04 to 0.20; *P*=0.188).

**Figure 2:**
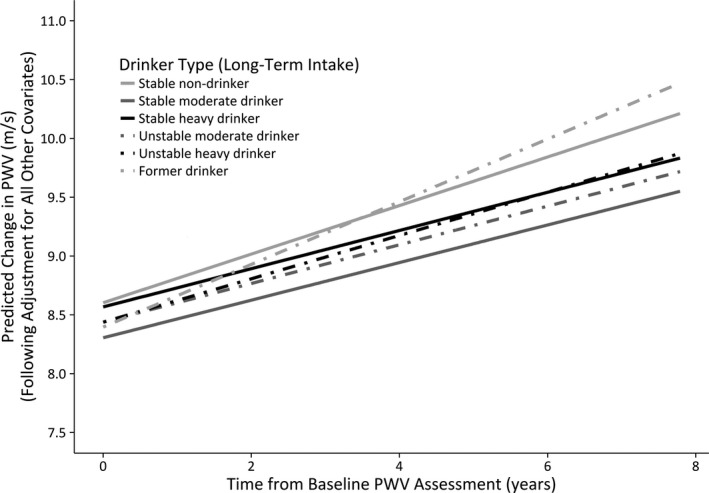
Male long‐term drinker types: changes in predicted PWV over time (maximally adjusted model). PWV indicates pulse wave velocity.

**Figure 3:**
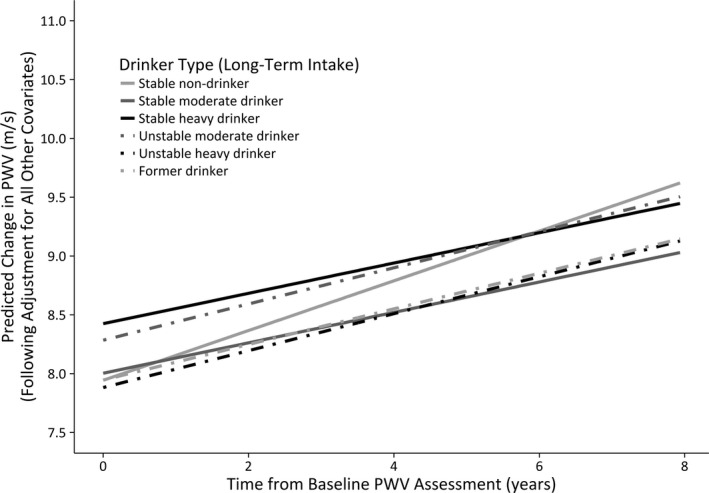
Female long‐term drinker types: changes in predicted PWV over time (maximally adjusted model). PWV indicates pulse wave velocity.

The predicted mean PWV trajectories, following maximal risk‐adjustment, are illustrated in Figures 2 and 3 for male and female participants, respectively. Figure 2 shows that among males, stable moderate drinkers have the lowest PWV values throughout the study period. Stable nondrinkers begin the study period as being the male drinker type with the highest PWV, but the plot illustrates that it is former drinkers who subsequently show the most accelerated increase in PWV among males over the follow‐up period. Figure 3 shows that stable heavy drinkers have the highest PWV at baseline among the female drinker types, but that it is stable nondrinkers who subsequently show the largest longitudinal change in PWV, albeit nonsignificantly.

A secondary aim of this study was to determine whether recent intake levels showed similar patterns of association to arterial stiffness. In the male and female samples, participants without any recent intake and those with recent heavy intake showed no significant difference in baseline PWV compared to recent moderate drinkers, regardless of the extent of covariate adjustment. In terms of longitudinal change, male participants with no recent reported intake had significantly higher PWV than those with recent moderate consumption (in the maximally adjusted model, b=0.07 m/s; 95% CI=0.01–0.13; *P*=0.021). No longitudinal effects were observed for the female sample. Results for the recent alcohol intake types are provided in Table 5.

**Table 5:** Effect of Recent Drinker Type on Baseline PWV and Longitudinal Changes in PWV: Sex‐Stratified Results

Model	Recent Drinker Type	PWV at Baseline^a^				Change in PWV (Per 4 Years)			
		Males		Females		Males		Females	
		Estimate (95% CI)	*P* Value	Estimate (95% CI)	*P* Value	Estimate (95% CI)	*P* Value	Estimate (95% CI)	*P* Value
Adjusted for age and assessment interval	No recent intake	0.10 (−0.16, 0.35)	0.446	−0.11 (−0.41, 0.19)	0.476	0.07 (0.01, 0.13)	0.019^b^	0.04 (−0.03, 0.11)	0.295
	Recent moderate drinker	Reference		Reference		Reference		Reference	
	Recent heavy drinker	0.10 (−0.07, 0.26)	0.247	−0.24 (−0.54, 0.05)	0.107	−0.01 (−0.05, 0.03)	0.605	0.03 (−0.04, 0.10)	0.437
As above with adjustment for demographics and lifestyle factors^c^	No recent intake	0.04 (−0.22, 0.29)	0.788	−0.20 (−0.51, 0.11)	0.202	0.07 (0.01, 0.13)	0.020^b^	0.04 (−0.03, 0.11)	0.302
	Recent moderate drinker	Reference		Reference		Reference		Reference	
	Recent heavy drinker	0.13 (−0.03, 0.30)	0.115	−0.17 (−0.47, 0.13)	0.274	−0.01 (−0.05, 0.03)	0.603	0.03 (−0.04, 0.10)	0.446
As above with adjustment for clinical factors^d^	No recent intake	0.06 (−0.18, 0.30)	0.621	−0.25 (−0.53, 0.03)	0.080	0.07 (0.01, 0.13)	0.021^b^	0.03 (−0.03, 0.10)	0.328
	Recent moderate drinker	Reference		Reference		Reference		Reference	
	Recent heavy drinker	0.09 (−0.07, 0.24)	0.259	−0.16 (−0.44, 0.12)	0.260	−0.01 (−0.05, 0.03)	0.569	0.03 (−0.04, 0.10)	0.413

PWV indicates pulse wave velocity.

a Unit of PWV is meters per second (m/s).

b Significant *P* values.

c Ethnicity, smoking, exercise, and socioeconomic position.

d Body mass index, heart rate, mean arterial pressure, diabetes mellitus, high‐density lipoprotein, and triglycerides.

As a sensitivity analysis following our use of multiple imputation, complete case analyses of the long‐term drinker type were undertaken. The same significant baseline and longitudinal associations were observed as in the imputed data analysis, with an additional significant baseline effect found for male stable nondrinkers. Full details of all models are available from the first author on request.

## Discussion

Using over 25 years of prospective alcohol consumption data, this study has provided new insights into the relationship between drinking and arterial stiffness. Male participants who habitually consumed heavy volumes of alcohol had significantly higher PWV than consistently moderate consumers (b=0.26 m/s; *P*=0.045). All drinker types, regardless of sex, experienced increases in their PWV from baseline across a subsequent 4‐ to 5‐year interval, but only male former drinkers showed significantly accelerated progression (b=0.11 m/s; *P*=0.009).

The finding that male stable heavy drinkers had higher baseline PWV than stable moderate consumers is consistent with existing research^9^, ^10^, ^11^, ^12^ and with the concept of a threshold effect for alcohol's impact on cardiovascular health.^42^ Compared to heavier volumes, moderate intake is known to be associated with higher high‐density lipoprotein cholesterol, a protective factor against arterial stiffening.^43^ A similar effect for stable heavy drinkers was observed among female participants after adjustment for demographic and lifestyle characteristics (b=0.73 m/s; *P*=0.029), but this was no longer significant after additional adjustment for clinical covariates (b=0.42; *P*=0.169). This difference from the male sample may be attributable to a disparity in statistical power because of the much smaller number of female stable heavy drinkers. However, it may also be attributable to sex differences in how the alcohol‐PWV relationship is confounded by other clinical characteristics. Increased high‐density lipoprotein has previously been shown to be associated with lower PWV in males, but not females, whereas triglycerides were a significant predictor of PWV in both sexes.^44^

There was also evidence in the female sample that unstable moderate drinkers had higher baseline PWV compared to the stable moderate group. This effect was again significant after adjustment for demographic and lifestyle characteristics (b=0.44 m/s; *P*=0.015), but not clinical covariates (b=0.28 m/s; *P*=0.091). Research into variable alcohol intake patterns has shown that triglyceride levels may attenuate the relationship between such variable intake levels and cardiovascular disease risk.^45^

When drinker type was defined according to recent intake only, the sole significant effect was for increased PWV progression among male nondrinkers (b=0.11 m/s; *P*=0.009). This association was also found by Matsumoto et al,^22^ the only previous study to measure both arterial stiffness and alcohol intake longitudinally. However, the alternative long‐term intake categorization used in the present study has enabled the differentiation of former drinkers from long‐term abstainers and shown that it is with former drinkers only that this longitudinal effect occurs. By capturing stability in intake levels over time, the current study has obtained new detail on the alcohol‐PWV relationship and addressed recent criticisms made of the wider alcohol epidemiology field.^46^

One‐off assessments can lead to misclassification of risk associations attributed to regression dilution.^47^ Comparing the long‐term and recent drinker types, 12% of male participants with moderate recent intake had predominantly drank heavily across the preceding 2 decades. Likewise, most recent male and female nondrinkers were former drinkers rather than long‐term abstainers (supplementary results are available from the first author on request). Only when longer‐term intake data are used to distinguish former drinkers from other participants in the recent intake analysis do we observe that former drinkers have a different risk of accelerated PWV change compared to the other drinker types. It is clear that the use of short‐term intake levels alone can potentially mask alcohol's association with cardiovascular risk.

In contrast to Matsumoto et al, no significant longitudinal effect was found here for heavy drinkers. Given the significant effect observed for male former drinkers, of whom fewer were sampled than heavy drinkers, it is unlikely this is attributable to power limitations. The baseline effect for stable heavy intake suggests that consistently heavy drinkers may experience more PWV change at a younger age compared to other drinker types. The significant longitudinal effect for former drinkers may partly be explained by the “sick‐quitter” phenomenon, where drinkers reduce their alcohol intake in response to the onset of ill health.^41^ This is substantiated by the finding that male former drinkers were significantly more likely to report poorer health than stable moderate consumers at the initial PWV assessment (supplementary results are available from the first author on request). No such difference was observed among the female participants, which is consistent with the absence of a longitudinal effect for female former drinkers.

This study endeavored to control for potential confounds of the association between alcohol and PWV. The finding that other risk factors for arterial stiffness, such as age, heart rate, and arterial pressure, are significantly associated with PWV in the direction anticipated^35^, ^36^, ^48^ provides additional confidence in our findings. The use of existing cohort data meant that covariate selection was dependent on available data and residual confounding may have occurred. Although most confounders of known relevance were captured in the current study, it may be useful to consider the potential impact of additional unmeasured confounders, such as statin usage and passive smoking, in future replications.

Regarding limitations, there was a possible selection bias given that only participants who remained in the Whitehall II study for over 2 decades took part in the PWV assessment. Similarly, because the consumption data covered a 25‐year timespan only, it is not possible to draw inferences about previous intake. Although our sample included consumers who consistently drank heavily, we may not have captured particularly high levels of consumption that could have had an incrementally greater impact on PWV.^25^ This was particularly true of the female sample, the analyses for whom had potentially reduced statistical power relative to the male‐only analyses. Alternative thresholds for defining lighter intake categories could also be explored where additional data are available. The current study's data are self‐reported and so vulnerable to estimation errors. These are issues known to affect, but not invalidate, population survey research.^49^ Regarding result generalizability, etiological evidence from the Whitehall II study has shown comparable results to community‐ and population‐based studies,^50^ and good concordance has been found between the prospective data collected in this cohort and retrospectively recalled drinking behavior.^51^ Additional evaluation of data reliability and validity would help verify this generalizability. PWV has to date only been assessed at 2 phases of the Whitehall II study, so neither nonlinear nor longer‐term changes in PWV could be tested. Finally, because of power limitations restricting further typology refinement, the current study was unable to incorporate other intake characteristics. Additional data may provide insights into other consumption patterns, such as episodic heavy drinking, which could further clarify the cardiovascular disease risk associated with unstable intake levels. Episodic heavy drinking could offset any theorized beneficial effect of moderate alcohol intake,^52^ indirect evidence of which is provided in the current study by the PWV differences between female stable and unstable moderate drinkers before adjustment for clinical factors. Limitations in the categorization approach used here, however, do not detract from our ability to draw comparisons to existing studies of alcohol and PWV, given that these typically also categorized drinking in terms of intake levels rather than frequency.

Bearing in mind these additional considerations, this study has furthered our understanding of how long‐term alcohol consumption is associated with arterial stiffness and its progression over time. This study has captured drinking profiles spanning over 2 decades and demonstrated the differing insights obtainable from cross‐sectional and longitudinal descriptions of drinking behavior. The study has shown that a consistently moderate drinking pattern is associated with lower arterial stiffness than is heavier drinking, particularly so among males. Discontinuation of drinking has also been shown to be associated in males with experiencing accelerated stiffness over time, and potential reasons for this and the absence of an effect among females have been offered. On the whole, the findings are compatible with the notion that consistently moderate alcohol intake is associated with lower cardiovascular risk, but suggest that the strength and form of this association may somewhat vary by sex. This work has, in particular, illustrated the new insights that can be obtained when the stability of intake levels is taken into account.

## Sources of Funding

The alcohol life‐course project (http://www.ucl.ac.uk/alcohol-lifecourse) is funded by the UK Medical Research Council/Alcohol Research UK (MR/M006638/1) and European Research Council (ERC‐StG‐2012‐309337_AlcoholLifecourse). The UK Medical Research Council (MR/K013351/1; G0902037), British Heart Foundation (RG/13/2/30098), and the National Institutes of Health (R01HL36310, R01AG013196) have supported the Whitehall II Study data collection.

## Disclosures

None.
